# Microbial Colonization Coordinates the Pathogenesis of a *Klebsiella pneumoniae* Infant Isolate

**DOI:** 10.1038/s41598-019-39887-8

**Published:** 2019-03-04

**Authors:** Jillian L. Pope, Ye Yang, Rachel C. Newsome, Wei Sun, Xiaolun Sun, Maria Ukhanova, Josef Neu, Jean-Pierre Issa, Volker Mai, Christian Jobin

**Affiliations:** 1 Department of Medicine, University of Florida, Gainesville, Florida USA; 2 Department of Epidemiology, University of Florida, Gainesville, Florida USA; 3 Department of Infectious Diseases and Pathology, College of Veterinary Medicine, University of Florida, Gainesville, Florida USA; 4 Department of Anatomy and Cell Biology, University of Florida, Gainesville, Florida USA; 5 Fels Institute for Cancer Research and Molecular Biology, Temple University, Philadelphia, PA 19140 USA; 6 Present Address: Department of Immunology & Microbial Disease, Albany Medical College, Albany, New York, USA; 7 Present Address: Department of Poultry Science, University of Arkanasas, Fayetteville, Arkansas USA

## Abstract

*Enterobacteriaceae* are among the first colonizers of neonate intestine. Members of this family, such as *Escherichia* and *Klebsiella*, are considered pathobionts and as such are capable of inducing local and systemic disease under specific colonization circumstances. Interplay between developing microbiota and pathogenic function of pathobionts are poorly understood. In this study, we investigate the functional interaction between various colonization patterns on an early colonizer, *K*. *pneumoniae*. *K*. *pneumoniae* 51-5 was isolated from stool of a healthy, premature infant, and found to contain the genotoxin island *pks* associated with development of colorectal cancer. Using intestinal epithelial cells, macrophages, and primary splenocytes, we demonstrate *K*. *pneumoniae* 51-5 upregulates expression of proinflammatory genes *in vitro*. Gnotobiotic experiments in *Il10*^−/−^ mice demonstrate the neonate isolate induces intestinal inflammation *in vivo*, with increased expression of proinflammatory genes. Regulation of microbiota assembly revealed *K*. *pneumoniae* 51-5 accelerates onset of inflammation in *Il10*^−/−^ mice, most significantly when microbiota is naturally acquired. Furthermore, *K*. *pneumoniae* 51-5 induces DNA damage and cell cycle arrest. Interestingly, *K*. *pneumoniae* 51-5 induced tumors in *Apc*^*Min*/+^; *Il10*^−/−^ mice was not significantly affected by absence of colibactin activating enzyme, ClbP. These findings demonstrate pathogenicity of infant *K*. *pneumoniae* isolate is sensitive to microbial colonization status.

## Introduction

Microbiota acquisition is a dynamic and progressive process, beginning from birth and gaining stability into the toddler stage of development^1,2^. The neonate gut is colonized through vertical transmission of maternal microbes, which can be influenced by factors including length of gestation^3^, mode of delivery (vaginal vs caesarean)^4^, and whether the infant is breast fed or formula fed^5,6^. The composition and diversity of the microbiota, particularly that of the intestine, is essential to the maintenance of host homeostasis. The gut microbiota demonstrates considerable influence on the development of host immunity^7^, and as such, perturbations of the microbiota composition can prove deleterious to the host. Interestingly, the intestinal microbiota of premature infants display high abundance of *Enterobactericiae*, primarily *Escherichia coli* and *Klebsiella pneumoniae*^8–10^.

Although microbiota composition can be dynamic within the first few years of life, “long-term colonizers” can stably occupy the intestinal tract. Their persistence has been associated with the expression of several virulence genes which may have deleterious consequence on the host. For example, *E*. *coli* belonging to the B2 phylogenetic group^11^, carry the pathogenic polyketide synthase (*pks*) island, a virulence gene cluster responsible for the production of colibactin, a genotoxin capable of inducing DNA damage^12^. The presence of the *pks* was strongly associated with the majority of long term colonizing *E*. *coli* strains identified in a longitudinal study involving infants^13^. Interestingly, we previously demonstrated a murine adherent-invasive *E coli* (NC101), containing the *pks*, contributes to the pathogenesis of colorectal cancer (CRC)^14^. Subsequent studies showed that *pks* + *E*.*coli* are present in biofilm of intestinal mucosal tissues from familial adenomatous polyposis (FAP) patients and participate, along with enterotoxic *Bacteroides fragilis* in carcinogenesis in pre-clinical models^15^. Other bacteria have also been discovered to carry the *pks* gene, including *Klebsiella pneumoniae*^12,16–18^, and exhibit cytotoxic capabilities *in vitro*^16,19^.

Similar to *E*. coli, *K*. *pneumoniae* is a rod shaped, facultative anaerobe, and a member of the Enterobactericiae family known to colonize the lung, oral cavity and intestine. Indeed, *K*. *pneumoniae* has been identified as a source of varied systemic and local infections^20^. Although most commonly associated with infection and inflammation of the lung^21,22^, there are reports that highlight an association between *Klebsiella* and intestinal maladies^23,24^. Mouse models of colitis have demonstrated inflammation can enhance the intestinal colonization of *K*. *pneumoniae*^25,26^, although *K*. *pneumoniae* colonization does not initiate disease. Additionally, there are few studies that report cooperation of *Klebsiella* with other microbes^25^ or chemical agents^27^ to enhance inflammatory reponse in preclinical models. To date, no study has shown an intestinal *Klebsiella* isolate could singly promote inflammation or carcinogenesis and whether colonization pattern influence this response.

With the use of gnotobiotic mice, we were able to simulate various colonization methods to investigate the pathogenic potential for the gut of a neonate isolate, *K*. *pneumoniae* 51-5. We demonstrate that a nonexistent and a slowly acquired microbiota, provide optimal conditions for *K*. *pneumoniae* 51-5 to induce intestinal inflammation and influence CRC.

## Results

### *K*. *pneumoniae* infant isolate induces inflammatory gene expression *in vitro*

A gram-negative bacterium (*K*. *pneumoniae* 51-5) was isolated from the stool of a premature infant. Using 16S sequencing and multi-locus sequence typing (MLST) analysis (Supplementary Table S1), we confirmed that the isolate belonged to the species *Klebsiella pneumoniae*, belonging to sequence type 1243 (ST1243).

**Figure 1:**
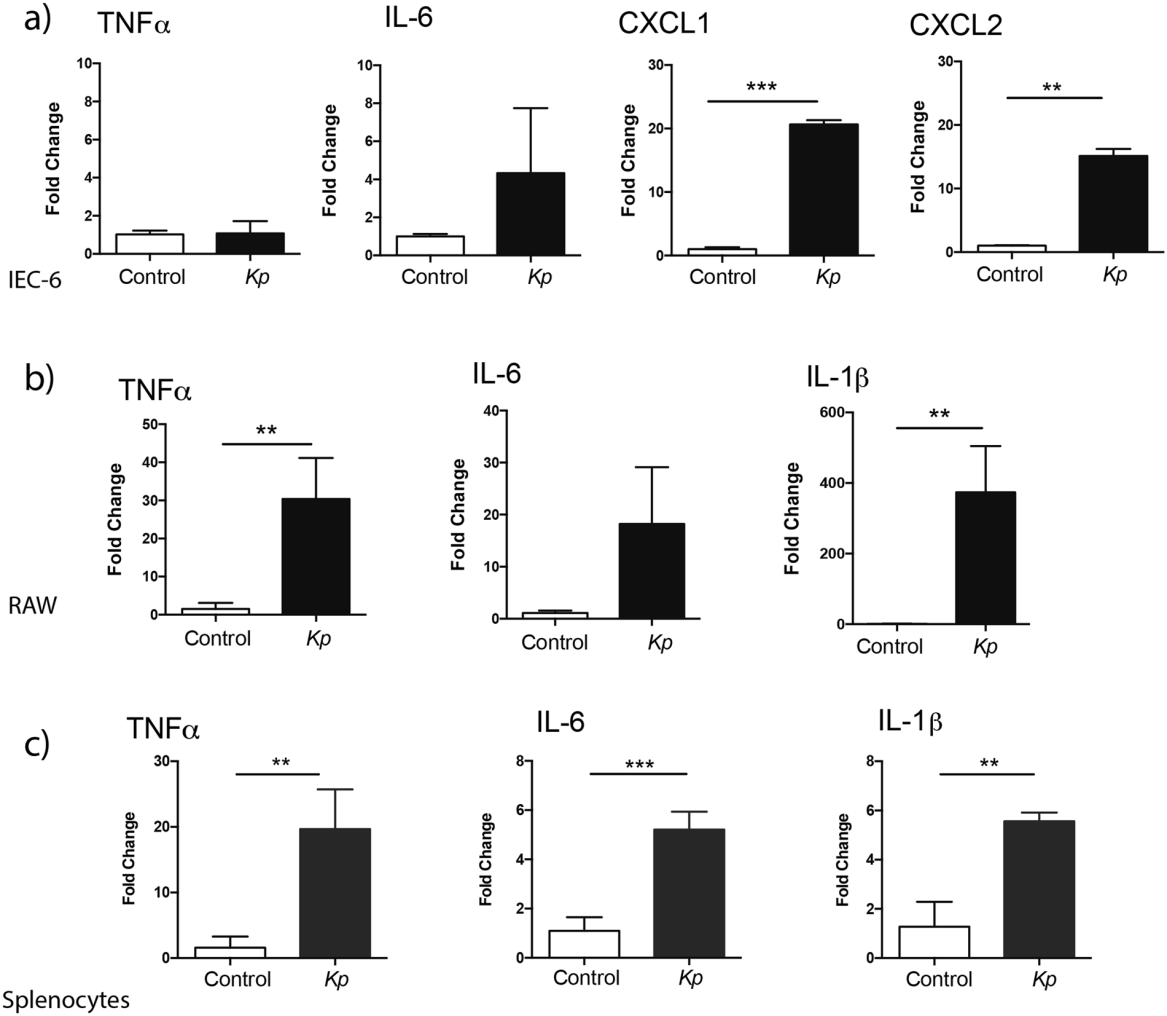
*K*. *pneumoniae* 51-5 infant isolate induces inflammation *in vitro*. Real-time PCR analysis of inflammatory cytokines and chemokines in (**a**) IEC-6 (**b**) RAW macrophages and (**c**) primary splenocytes infected with *K*. *pneumoniae (Kp)*. Results are representative of at least three independent experiments performed in triplicates. Graphs represent means ± SD. **P < 0.01; ***P < 0.001 (Unpaired student t-test, Welch’s correction).

To assess the inflammatory potential of our *K*. *pneumoniae* 51-5 infant isolate, we infected intestinal epithelial cells (IEC) and immune cells and examined cytokine mRNA expression using real-time PCR. We observed a significant upregulation of *Cxcl1* (p = 0.0077, p = 0.0005) and *Cxcl2* (p = 0.0019, p = 0.0002) mRNA in *K*. *pneumoniae* infected IEC-6 cells (Fig. 1a) and in Mode K cells (Supplementary Fig. S1), respectively, with no significant changes in *Tnfα* or *Il6* mRNA levels. In addition, a significant upregulation of *Tnfα* (p = 0.0407), *Il-1β* (p = 0.0388), and marked increase in *Il-6* (p = 0.0529) mRNA transcripts, compared to uninfected controls, was observed in immune RAW cells (Fig. 1b). Finally, primary murine splenocytes infected with *K*. *pneumoniae* 51-5 showed increased expression of *Tnfα* (p = 0.0069), *Il-6* (p = 0.0002), and *Il-1β* (p = 0.0018) mRNA (Fig. 1c). These data demonstrate the potential of the infant *K*. *pneumoniae* isolate to trigger an inflammatory response in both IECs and immune cells.

### *K. pneumoniae* monoassociation induces severe colitis

**Figure 2:**
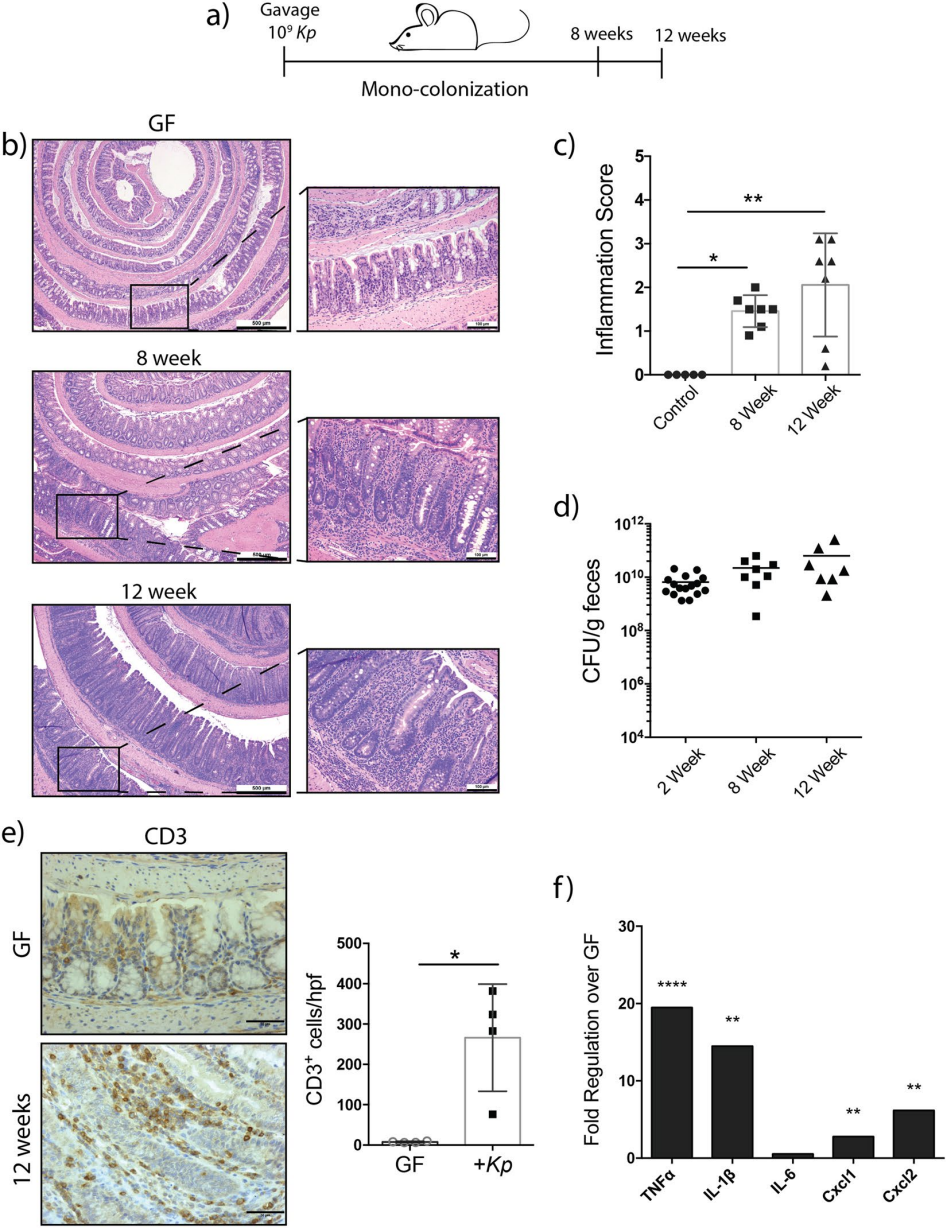
Monoassociation of *K*. *pneumoniae* 51-5 in germ-free *Il10*^−/−^ mice. (**a**) Experiment setup for mono-colonization. (**b**) Representative H&E staining of GF mice (n = 5) infected with *Kp* for 8 and 12 weeks (n = 7) were scored (**c**) for inflammation (ANOVA, Kruskal-Wallis with Dunn’s post-test). (**d**) Bacterial colonization confirmed by fecal plating at 2, 8, and 12 weeks. (**e**) CD3 immunostaining and quantification of GF and 12 week infected colon tissue. Quantification of 10 hpf each of 4 mice per group. (Mann-Whitney t-test). (**f**) Cytokine qPCR array of 12 week *K*. *pneumoniae* 51-5 *-*infected mice compared to GF. Graphs represent means ± SD. *P < 0.05; **P < 0.01; ****P < 0.0001. Scale bars in bottom right corner of images are 500 μm (**b**) and 50 μm (**e**); 100 μm insets.

To assess the inflammatory potential of *K*. *pneumoniae* 51-5 *in vivo*, we performed mono-association studies in germ-free (GF) *Il10*^−/−^ mice (129SvEv), a colitis model susceptible to bacteria colonization. Mice were orally gavaged with *K*. *pneumoniae* 51-5 (10^9^ CFU/mice) and maintained in gnotobiotic isolators for 8 and 12 weeks (Fig. 2). Successful colonization was confirmed measuring viable bacteria in reconstituted feces (Fig. 2d). Histological assessment revealed a significant increase of inflammation in mono-colonized mice compared to GF *Il10*^−/−^ controls (p = 0.0421) at 8 weeks, mostly localized to proximal region of the colon. Histological presentation of the small intestine of *K*. *pneumoniae* 51-5 infected mice was similar to those of GF controls, suggesting a colon-specific inflammatory effect of the bacterium (data not shown). After 12 weeks, mono-colonized mice showed development of pan-colitis and significant increase in inflammation (p = 0.0022 vs GF control), with marked hyperproliferation present throughout the colon, particularly in the distal regions (Fig. 2b).

Immunohistochemical analysis revealed a significant increase in CD3 + cells per field (p = 0.0286) in the lamina propria of 12 week mono-colonized mice, suggesting lymphocyte and myeloid cell infiltration in the intestine (Fig. 2e). In accordance with histological results, a significant increase in *Tnfα* (19-fold, p = 0.000068) and *Il-1β* (14-fold, p = 0.009617), and mRNA accumulation was observed in mono-colonized mice compared to GF mice (Figs 2f and S2). Additionally, elevated levels of innate immune regulators, *Cxcl1* (3-fold, p = 0.006853) and *Cxcl2* (6-fold, p = 0.002862) mRNA were also detected. Mediators of bacterial defense, *Ccl8* (110-fold, p = 0.001097), *Cxcl9* (35-fold, p = 0.005890), and *Nos2* (16-fold, p = 0.000038) mRNA were also significantly upregulated in *K*. *pneumoniae*-infected mice compared to controls (Supplementary Fig. S2). Taken together, these results demonstrate the *K*. *pneumoniae* infant isolate has the capacity to induce colitis *in vivo* without the requirement of a complex biota.

### Microbiota assembly regulates *K*. *pneumoniae*-induced intestinal inflammation

**Figure 3:**
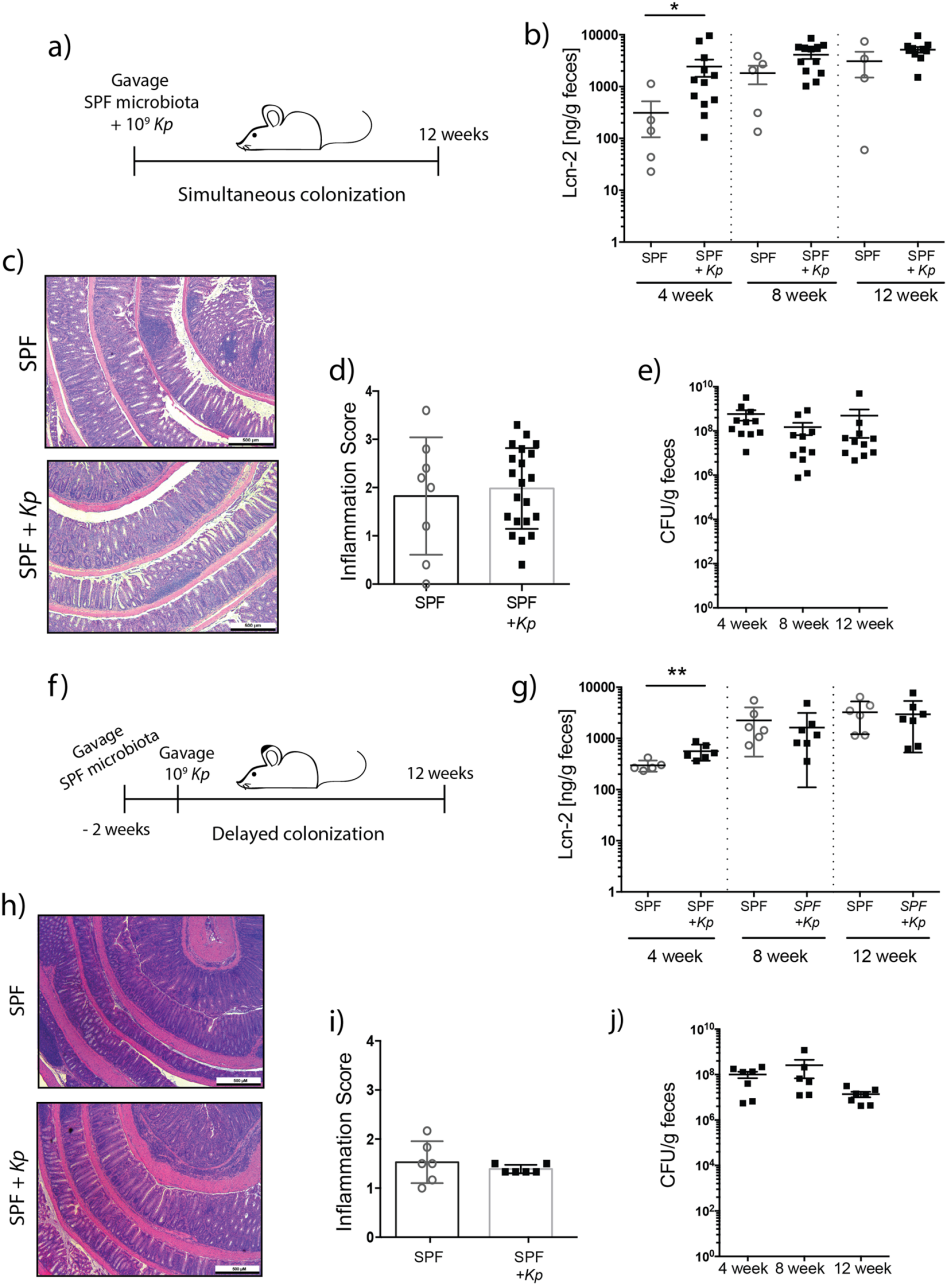
*K*. *pneumoniae* 51-5 accelerates the onset of inflammation. (**a**) Experiment setup for simultaneous colonization. (**b**) Fecal Lcn-2 ELISA of mice at 4, 8, and 12 weeks. (**c**) H&E stained colon from representative control and SPF + *Kp* gavaged mice after 12 weeks. (**d**) Colonic inflammation score from SPF control (n = 8) and SPF + *Kp* (n = 21) mice combined from 2 separate experiments. (**e**) Experiment setup for delayed colonization. (**f**) Fecal Lcn-2 of control and mice gavaged with *Kp* after 4, 8, and 12 weeks. (**g**) H&E stained colon from representative control and delayed *Kp* gavaged mice after 12 weeks. (**h**) Colonic inflammation score of control (n = 6) and delayed *Kp* gavaged mice (n = 6) after 12 weeks. Graphs represent mean ± SD. Scale bars in bottom right corner of images are 500 μm. *P < 0.05; **P < 0.01 (Mann-Whitney t-test).

We next tested whether *K*. *pneumoniae* 51-5 can induce colitis in the presence of a complex and diverse microbiota using a co-colonization model (Fig. 3a).

In this simultaneous colonization model, GF *Il10*^−/−^ mice were colonized for 12 weeks by single oral gavage with specific pathogen free (SPF) microbiota obtained from the ceca of wild type mice alone or in combination with *K*. *pneumoniae* 51-5 (SPF + *Kp)*. We measured fecal lipocalin 2 (Lcn-2), a sensitive marker of inflammation, through the course of the experiment. *K*. *pneumoniae-*infected mice produced a significantly higher amount of Lcn-2 (p = 0.0136) at an early timepoint (4 weeks) (Fig. 3b). Interestingly, the addition of *K*. *pneumoniae* 51-5 did not have a synergistic effect on intestinal inflammation as assessed by H&E (Fig. 3c) and mean inflammation score (1.98 vs 1.89) (Fig. 3d) after 12 weeks. To determine whether the infant isolate was excluded due to additional bacteria of the SPF inoculum, we plated reconstituted stool to determine bacterial colonization. The presence of the complex biota did not impact *K*. *pneumoniae* 51-5 colonization level over the course of the experiment (Fig. 3e). No significant difference was observed in immune infiltration by CD3 immunostaining nor cytokine (*Tnfα*, *Il-6*, *Cxcl2)* mRNA accumulation between cohorts, although *Il1β* was significantly increased in *K*. *pneumoniae* colonized mice (Supplementary Fig. S3).

To determine whether *K*. *pneumoniae* 51-5 can initiate inflammation in mice with a pre-established microbiota, we used a delayed colonization model where SPF biota was introduced by oral gavage 2 weeks prior to *K*. *pneumoniae* infection (Fig. 3f). In this model, a significant increase in Lcn-2 (p = 0.0087) production was also observed with the addition of *K*. *pneumoniae* 51-5 after 4 weeks compared to controls (Fig. 3g). Similar to the previous model, we observed a loss of this separation over time, with no significant difference in histology (Fig. 3h) or inflammation score after 12 weeks (Fig. 3i), suggesting *K*. *pneumoniae* plays an important role in the onset of inflammation.

**Figure 4:**
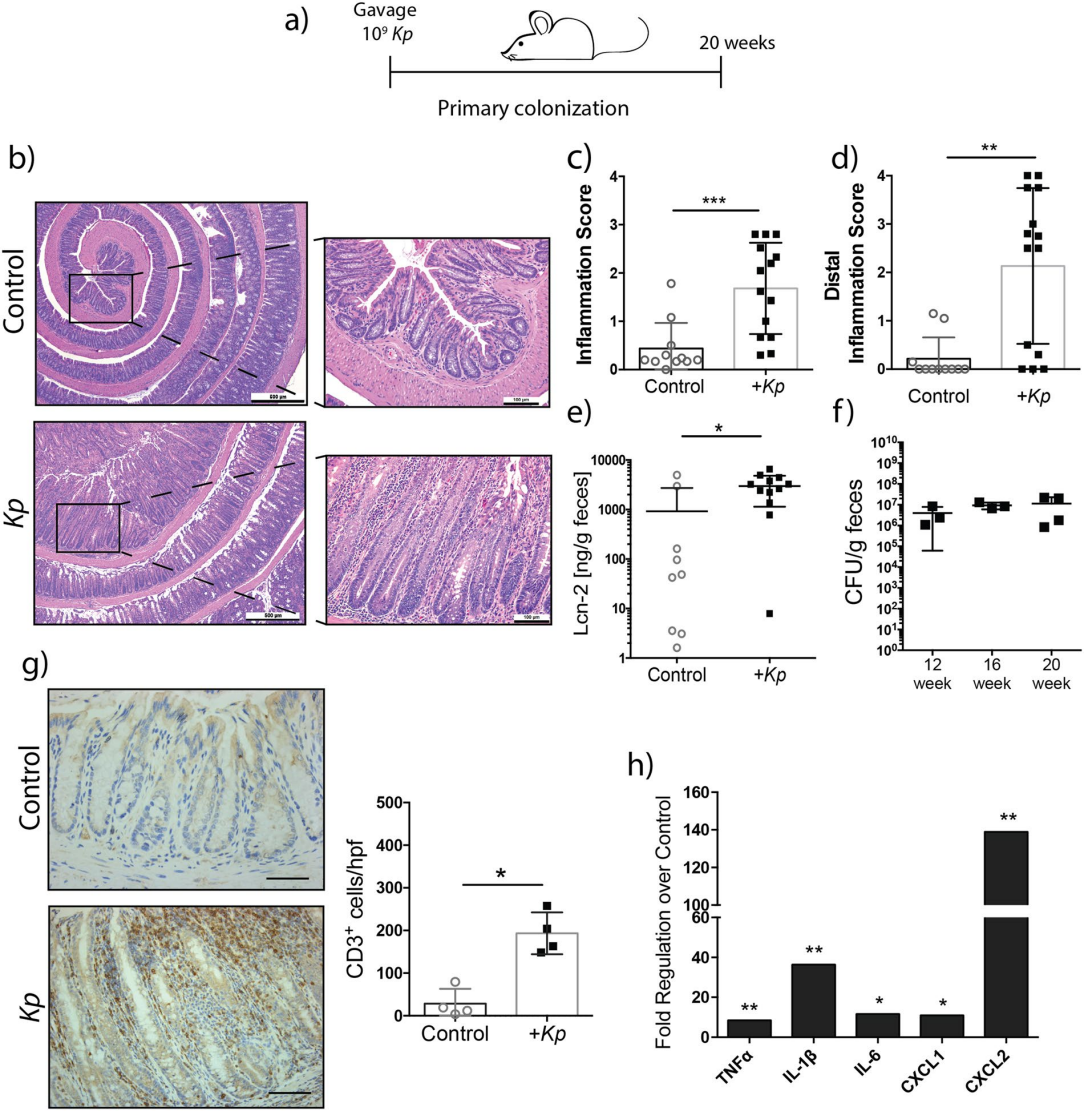
Long term colonization of *K*. *pneumoniae* 51-5 induces severe colitis with naturally acquired microbiota. (**a**) Experiment setup. (**b**) Representative H&E stained colons of control (n = 11) and 20 week infected mice (n = 14). (**c**) Total inflammation score of control and infected colons and (**d**) distal colonic tissue, combination of two experiments. (**e**) Representative CD3 staining and quantification, n = 4, quantification 10 hpf per group. (**f**) Cytokine qPCR array of colons from control and infected mice. Graphs represent mean ± SD. *P < 0.05; **P < 0.001; ***P < 0.001. Scale bars in bottom right corner of images are 500 μm (**b**) and 100 μm (**g**); Insets are 100 μm.

To examine the influence of microbiota assembly on host susceptibility to *K*. *pneumoniae* infection, we used a primary colonization model where GF *Il10*^−/−^ mice were transferred to SPF housing and immediately colonized with *Klebsialla* 51-5 by oral gavage (Figs 4a and S4). In this model, control (no gavage) and *K*. *pneumoniae* associated mice were allowed to naturally acquire an intestinal microbiota through husbandry environmental exposure for 20 weeks. Mice were sacrified at 8 and 20 weeks following transfer to assess intestinal inflammation at early and late time points respectively. After 8 weeks, we observed a significant increase in inflammation (p = 0.00376), particularly in the distal colon (p = 0.0263) with a significant increase in number of CD3-positive immunostained cells (p = 0.0130) (Supplementary Fig. S4). *K*. *pneumoniae*-infected mice demonstrated significantly higher Lcn-2 production as early as 4 weeks post-infection (p = 0.0051) that was sustained to 8 weeks (p = 0.0059) (Supplementary Fig. S4g). A significant increase of proinflammatory cytokines *Tnfα* (p = 0.0159), *Il-6* (p = 0.0159), *Cxcl2* (p = 0.0357) and *Il1-β* (0.0159) mRNA was also detected in colonic tissues of *K*. *pneumoniae*-infected mice after 8 weeks colonization (Supplementary Fig. S4h). Following 20 weeks of colonization, *K*. *pneumoniae*-associated mice demonstrated increased colonic hyperplasia (Fig. 4b) and a significant increase in immune infiltration as assessed by CD3 immunostaining (p = 0.0286) (Fig. 4g), which was confirmed by increased inflammation score (p = 0.0002) (Fig. 4c). Much of this inflammation was localized to the distal colon (Fig. 4d) of infected mice (p = 0.0021). A significant increase in fecal lipocalin (p = 0.0184), cytokine (*Tnfα* (p = 0.00241), *Il1-β* (p = 0.00245), *Il-6* (p = 0.04004)), and chemokine (*Cxcl1* (p = 0.03583) and *Cxcl2* (p = 0.00603)) mRNA accumulation were observed in *K*. *pneumoniae*-infected mice compared to controls (Figs 4e,h and S5).

### Genotoxic *K*. *pneumoniae* contributes to colitis-associated tumorigenesis

**Figure 5:**
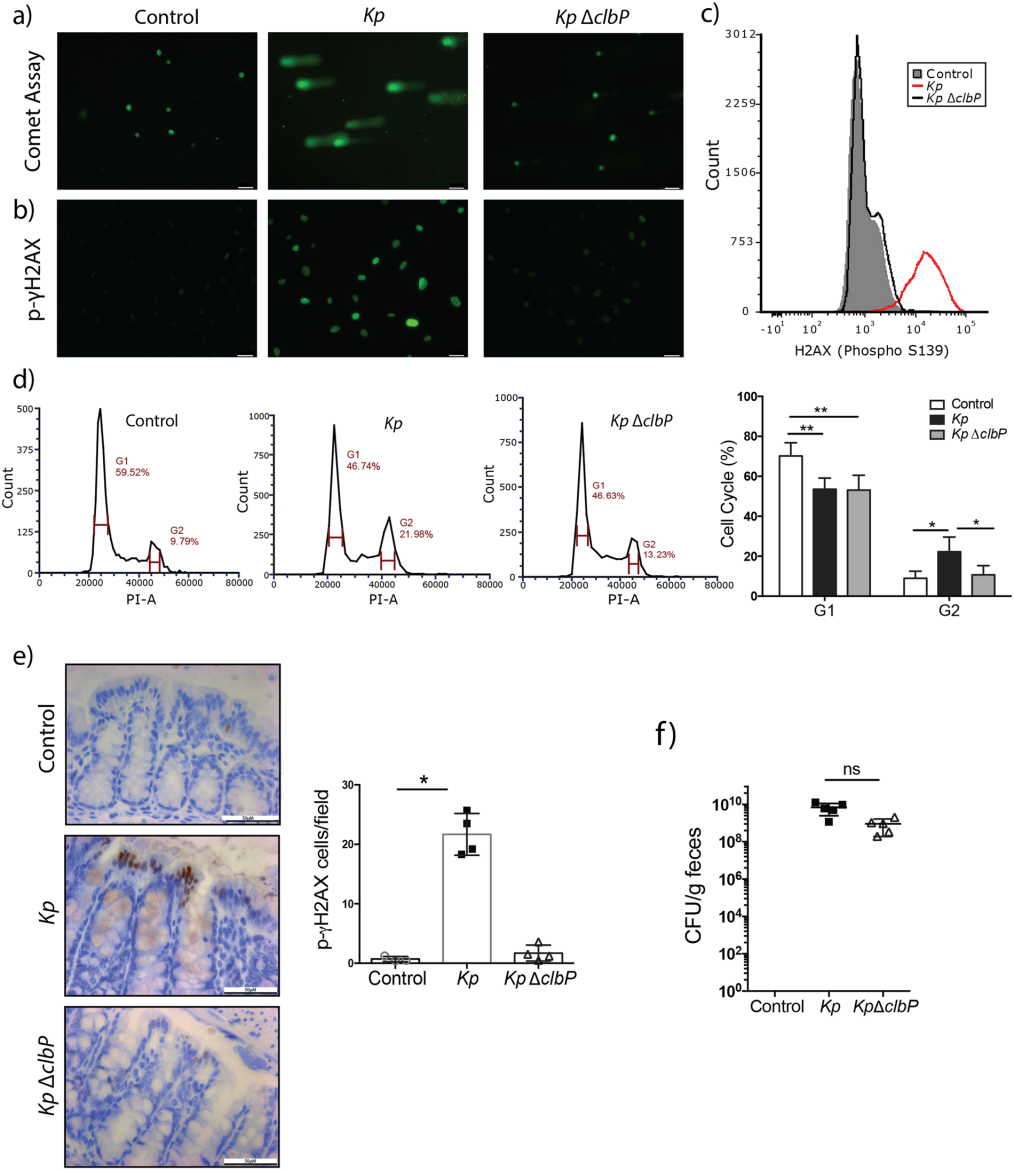
Infant Isolate *K*. *pnenumoniae* 51-5 induces DNA damage in intestinal epithelial cells. (**a**) Representative fluorescent images of DNA damage measured via comet assay and (**b**) phospho-γH2AX staining in control and infected IEC-6 cells. Flow cytometry anlaysis of Phospho H2AX stained cells (**c**) and cell cycle analysis (**d**) of control, *Kp*, and *Kp*ΔclbP*-*infected IEC6 cells displayed on linear scale. Histograms are a representative of 3-4 individual experiments. Quantification of cell cycle mean of 3–4 experiments. (2 way ANOVA, Tukey’s multiple comparison). (**e**) Representative images of mouse colon after 1 week infection, stained with phospho-γH2AX and quantification. Graph represents quantification of 10 fields per mouse, of 3–4 mice per group. (One way ANOVA) (**f**) Colonization of 1 week mice infected with *K*. *pneumoniae* 51–5 and *K*. *pneumoniae* 51-5 ΔclbP compared to control (no detectable colonies), ANOVA. Graphs represent mean ± SD. ns,not significant. *P < 0.05; **P < 0.001 Scale bars in bottom right corner of images are 60 μm (**a**,**b**) and 50 μm (**e**).

As previously mentioned, the *pks* island is a conserved sequence found among select bacteria and identified as the source of cellular DNA damage. Using primers designed for the flanking regions of the *pks* in *E*. *coli*, we detected the presence of the *pks* island in the genome of our infant *K*. *pneumoniae* isolate, suggesting that this strain could exert a genotoxic effect (Supplementary Fig. S6). Indeed, functional assays showed *K*. *pneumoniae* 51-5 infection increased DNA damage in IEC-6 cells as determined by increased comet tails and γH2AX immunofluorescence (Fig. 5a–c). *K*. *pneumoniae*-infected cells also demonstrated significantly increased (p = 0.0156) cell numbers in the G2 phase (Fig. 5d). To determine whether the *pks* was necessary for DNA damage induced by our isolate, we deleted the *clbP* gene which encodes for ClbP enzyme, a peptidase responsible for the release of mature toxin^28,29^ (Supplementary Fig. S6). Infection with isogenic *clbP* mutant (*Kp* ΔclbP) was sufficient to abrogate DNA damage as observed by reduction of comet tails, decreased γH2AX immunofluorescence and cell cycle arrest (p = 0.0364) (Fig. 5a–d). We then tested the *in vivo* genotoxic potential of *K*. *pneumoniae* 51-5 by colonizing ex-GF *Il10*^−/−^ mice for seven days and examining γH2AX production (Fig. 5e). The colon of *K*. *pneumoniae* 51-5 infected mice showed increased staining for γH2AX compared to uninfected (p = 0.0202) or *K*. *pneumoniae* 51-5 ΔclbP infected mice (Fig. 5e). Bacterial colonization of *Kp* ΔclbP was confirmed by stool plating to enumerate CFU, with no significant change compared to *K*. *pneumoniae-*infected mice (Fig. 5f). These results confirm the genotoxic capability of the *K*. *pneumoniae* neonate isolate.

**Figure 6:**
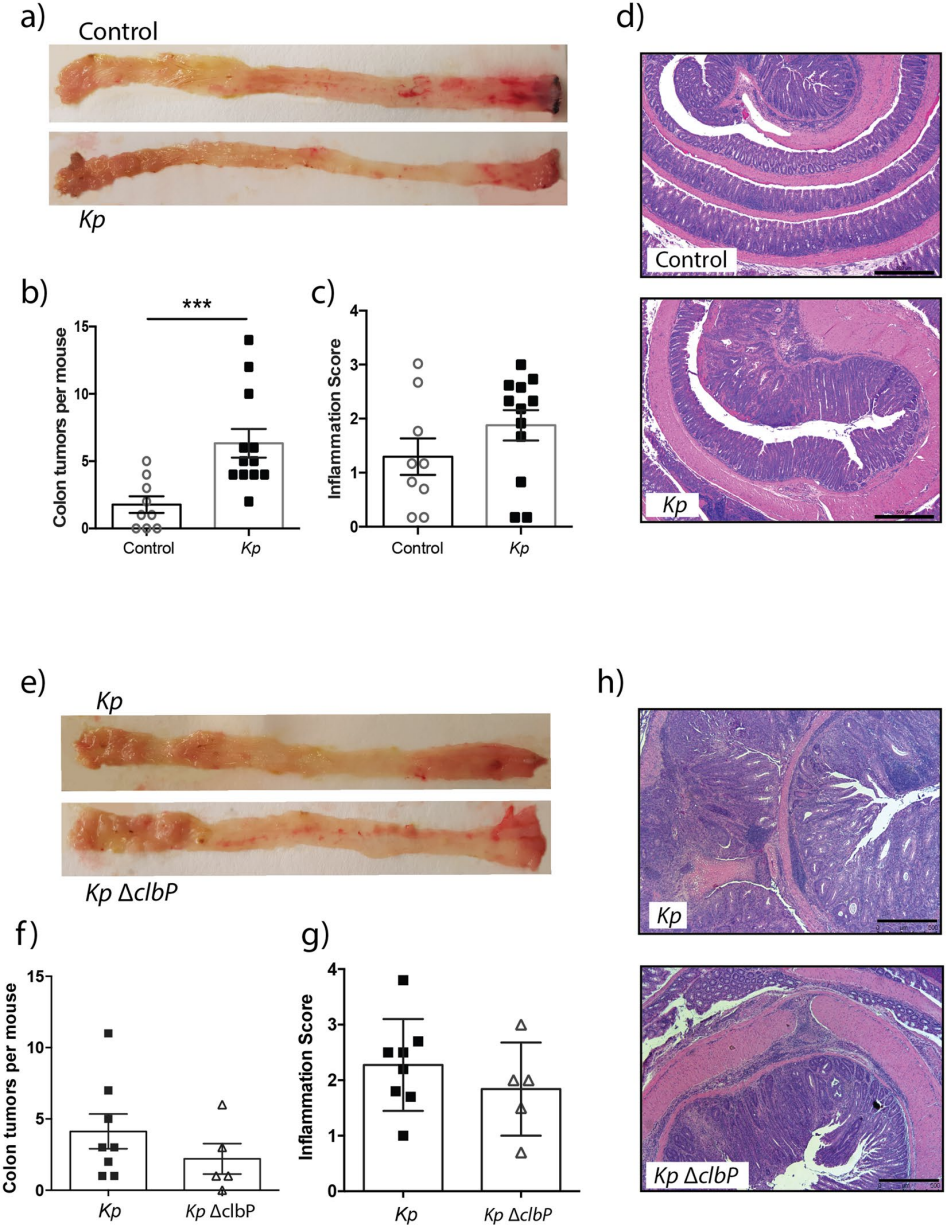
*K*. *pneumoniae* 51-5 increases colonic tumors in model of colitis-associated cancer independent of *clbP*. (**a**) Representative macroscopic pictures of mouse colon and (**b**) quantification of tumors from control (n = 9) and *Kp* infected *Apc*^*Min*/+^; *Il10*^−/−^ mice (n = 12) at 20 weeks. (**c**) Inflammation score and (**d**) representative H&E stained colon tissue. (**e**) Representative macroscopic pictures of mouse colon and (**f**) quantification of colonic tumors from *Kp* (n = 8) and *Kp*ΔclbP infected (n = 5) *Apc*^*Min*/+^; *Il10*^−/−^ mice at 20 weeks. (**g**) Inflammation score (**h**), and representative H&E images of *Kp* and *Kp*ΔclbP infected mice. Data represented are 1 of 2 independent experiments. Graphs represent mean ± SD. ***P < 0.001. Scale bars in bottom right corner of images are 500 μm (**d**,**h**).

We recently published a novel model of spontaneous colitis-associated tumorigenesis where GF *Apc*^*Min*/+^; *Il10*^−/−^ mice developed colonic tumors when colonized with specific microorganisms^30^. To determine the pro-tumorigenic capacity of *Klebsiella* 51-5, we transferred GF *Apc*^*Min*/+^; *Il10*^−/−^ mice to SPF housing and colonized them with *K*. *pneumoniae* 51-5 via oral gavage. After 20 weeks of colonization, *K*. *pneumoniae*-infected mice demonstrated a significant increase (p = 0.0009) in the number of colonic tumors (Fig. 6a,b), with no significant increase in inflammation (Fig. 6c,d).

To assess the impact of colibactin deficiency on tumorigenesis we infected *Apc*^*Min*/+^; *Il10*^−/−^ mice with *K*. *pneumoniae* 51-5 ΔclbP or *K*. *pneumoniae* 51-5. While *K*. *pneumoniae* ΔclbP mice presented with fewer colon tumors compared to *K*. *pneumoniae*-infected mice, these differences were not statistically significant (Fig. 6e,f). Additionally, there was no significant difference detected in the level of inflammation between cohorts (Fig. 6g,h). While ClbP is essential for genotoxicity, it does not seem to significantly affect the pro-carcinogenic potential of *K*. *pneumoniae* 51-5.

Overall, our data reveal that *K*. *pneumoniae* 51-5 mediates intestinal pathologies which is sensitive to microbiota status.

## Discussion

Enterobacteriaceae are among the early bacterial colonizers of the lower intestinal tract, and the relative abundance of some genera from this family such as *Escherichia* and *Klebsiella* are often increased in intestinal pathologies including IBD, NEC and CRC^23,24,31,32^. Although the colitogenic potential of some strains of *E*. *coli* has been demonstrated in various gnotobiotic conditions^33^, the relationship between *K*. *pneumoniae* and colitis is still unclear. Here, we demonstrated that a *K*. *pneumoniae* infant isolate, *K*. *pneumoniae* 51-5, has the ability to promote intestinal inflammation in a host lacking the immunosuppressive cytokine Il10. This colitogenic property is observed in mono-associated and in naturally colonized *Il10*^−/−^ mice, but diminished in mice colonized with a full complement of microbiota. Since defective Il10 signaling has been associated with very early onset IBD^34–36^, interactions between environment, such as early bacterial colonization and host genetics, is likely a critical component of intestinal pathology.

To determine interaction between microbiota and colitogenic potential of *K*. *pneumoniae* 51-5, we utilized a varied set of microbial colonization models employing a gnotobiotic approach with *Il10*^−/−^ mice. We first mono-associated *Il10*^−/−^ mice with *K*. *pneumoniae* 51-5. We observed significant hyperplasia and immune cell invasion response throughout the colon after 12 weeks of colonization, suggesting this *K*. *pneumoniae* isolate is an alpha bacterium in this model. Interestingly, in the presence of a complex microbiota, *K*. *pneumoniae* colonization had no synergistic effect on inflammation, despite maintaining a high colonization level. Temporal analysis of Lcn-2 production allowed us to conclude that *K*. *pneumoniae* infection may play a role in the early stages of inflammation, regardless of microbial status. This supports previously published reports that utilize the addition of *K*. *pneumoniae* to accelerate inflammation in mouse models of intestinal disease^27,37^. In contrast, when GF mice acquired microbiota through environmental exposure (natural acquisition), *K*. *pneumoniae* colonization resulted in a strong inflammatory response compared to control mice. These experimental groups had comparable levels of *K*. *pneumoniae* colonization suggesting the attenuated inflammation observed in the SPF + *K*. *pneumoniae* 51-5 and the naturally colonized mice + *K*. *pneumoniae* 51-5 cohorts is not due to exclusion of the bacterium from the intestine. The extent of inflammation observed between these groups could be explained by the early interactions between *K*. *pneumoniae* 51-5 and resident microbes at the time of infection. We previously reported that natural acquisition of microbiota from the environment results in an initial varied composition of bacterial communities compared to SPF gavaged mice, that becomes more comparable between the 4 and 8 week timepoint after colonization^38^. The effects of *K*. *pneumoniae* on inflammation has been most successfully studied within the context of lung infections, an environment initially thought to be sterile. Recent identification of an oral isolate of *K*. *pneumoniae* inducing colonic inflammation was performed under germ free or antibiotic conditions^39^. Our data corroborates these findings in that mice devoid of bacteria are highly susceptible to *K*.*pneumoniae* infection, but also introduces another concept where a shift in the relative amounts of certain bacterial communities, through modulation of microbiota assembly, can regulate its colitogenic effects. *K*. *pneumoniae* belongs to the Proteobacteria phyla which is known to predominate in the immature gut^40^ and a decrease in this community is associated with maturation of the host biome. Indeed we have previously demonstrated a decrease in Proteobacteria abundance with time after conventionalization of germ free mice by gavage or natural acquisition^38^. It is possible that gavage with *K*. *pnemoniae* 51-5 can influence the microbiota development by sustaining a high level of Proteobacteria which could influence pathogenesis of this isolate. Further investigation using taxonomic analysis (16S rDNA sequencing) will be needed to determine impact of early gavage of *K*. *pneumoniae* on host microbiota composition.

Host genetics also play a crucial role in microbial pathogenesis. For example, *K*. *pneumoniae* was one of the bacterial species enriched in the model of *T-bet*^−/−^;*Rag2*^−/−^ ulerative colitis (TRUC)^25^. However the bacterium was unable to induce disease on its own when introduced to GF TRUC mice^25^. When introduced to a WT or *Rag2*^−/−^ mouse, *K*. *pneumoniae*, in concert with TRUC resident microbes, was able to elicit inflammation^25^. While this response may be specific to the isolate, a possible mechanism could be due to unnoticed defects in Th1 signalling that may be intrinsic to TRUC. Recently, an oral isolate was demonstrated to elicit colonic inflammation in GF *Il10*^−/−^ model via Th1 immune signaling, particularly *Ifn*γ^+^ T cells^39^. While we observed significant upregulation of *Ifn*γ via cytokine array (Supplementary Fig. S2 and S5), further analysis is needed to confirm regulation through Th1 signalling in our models.

Sequencing analysis identified a high relative abundance of *K*. *pneumoniae* within the mucosal sites of CRC patients^23^ however the functional role of this bacterium in CRC is unknown. The presence of the pathogenic *pks* island, responsible for the production of the genotoxin colibactin, has been identified in Western CRC patients and is critical for *E*. *coli*-induced tumor formation in mice^14,41^. We demonstrated that an active colibactin is generated from the infant isolate *K*. *pneumoniae* 51-5 since genetic ablation of the *clbP* gene reduces DNA damage and cell cycle arrest in cell lines. Surprisingly, *K*. pneumoniae-induced CRC in *Apc*^*Min*/+^; *Il10*^−/−^ mice was not significantly attenuated when colibactin is functionally disrupted, suggesting that other mechanisms contribute to carcinogenesis. It is important to note the full length *pks* gene cluster was not deleted in our experiments, and as such, there remains several *Clb* genes whose functions extend beyond colibactin production. For example, *clbS* encodes for an anti-toxin^42,43^, *clbM* produces a xenobiotic transporter^44,45^ while *clbA* participates in the production of iron scavenger siderophores by Enterobactin (*Ent*) and yersiniabactin (*Ybt*) found in the High-Pathogenicity Island (HPI) loci^46^. Dual loss of *Ent* and *Ybt* is sufficient to reduce cytokine production and *Klebsiella* colonization in lung homogenates^22^. In addition, deletion of the *clbA* gene from *K*. *pneumoniae* sufficiently reduced cytokine production and bacterial colonization in a murine model of meningitis^47^. It is also noteworthy that *K*. *pneumoniae* contains several virulence factors demonstrated to affect colonization, immune cell recruitment, and inflammation^47–49^. One example is the fimbrial adhesion gene, *fimK*, which functions in host cell attachment, and shown to be critical for lung colonization and proinflammatory response^48^. Further studies would be needed to address the function of *pks* island and other virulent genes in *K*. *pneumoniae* 51-5.

In summary, we have demonstrated early acquisition of an infant isolate of *K*. *pneumoniae*, is sufficient to induce inflammation in a microbe-free environment or immature nascent microbiota, while the presence of a complex biota attenuates this response.

## Methods

### Ethics statement

All experiments were performed in accordance with relevant guidelines and regulations. Written informed consent was obtained from the parent(s) of the infant and in accordance with the principles expressed in the Declaration of Helsinki. The study, including consent procedure was approved by the UF Health Institutional Review Board 01.

### Isolation and characterization of the neonate *Klebsiella pneumoniae* clinical isolate

The *K*. *pneumoniae* 51-5 isolate was obtained from a frozen or glycerol preserved sample from Mac Conkey agar by screening for lac + colonies that lack motility. Identity of the isolated strain was then confirmed via Sanger sequencing of the 16S rRNA gene and qPCR for *K*. *pneumoniae* specfic genes, *gltA*^50^ and *ITS*^51^. Initial characterization using standard microbiological methods resulted in positive test result of the isolate for urease test, negative for indole test, and negative for gram staining (*data not shown)*. The presence of the genotoxic *pks* island was determined by PCR using primers specific for flanking regions of the *pks* island (R1 and R2) as described previously^14^. Finally, we performed a Multi-Locus Sequence Typing (MLST) Analysis on our *K*. *pneumoniae* 51-5 using seven house keeping genes commonly used to confirm the identity of *Klebsiella pneumoniae* isolates^52^. Briefly, genomic DNA was isolated from an overnight culture of *K*. *pneumoniae* 51-5. Using the primers provided by the University of Oxford PubMLST^53^ (https://pubmlst.org/kpneumoniae/info/primers.shtml), genes were amplified by PCR, gel purified (Qiagen Mini-Elute Gel Extraction) and submitted for sequencing. Forward and reverse sequences were quality trimmed and joined using the Staden package^54^ (http://staden.sourceforge.net) to generate a consensus sequence for each locus. The consensus sequence was then aligned to the reference sequence from PubMLST using the online NCBI BLAST align two sequences program (accessed on October 29, 2018) to generate the percent sequence identity between the our consequence and the full length PubMLST reference. The sequences were also submitted to the Institute Pasteur https://bigsdb.pasteur.fr/klebsiella/klebsiella.html to identify the sequence type.

### Generation of isogenic Δ*clbP* mutant

All bacterial strains and plasmids generated in this study are listed in Supplementary Table S2. All strains were stored at −70 °C in peptone-glycerol. *E*. *coli* Top 10 was used as an intermediate host for cloning procedures and grown routinely at 37 °C in LB broth^55^ or on LB solidified with 1.2% Bacto Agar (Difco). The *K*. *pneumoniae* strain used in this study was grown in LB medium at 37 °C. When required, chloramphenicol (30 μg/ml, Cm) was added. LB containing 8% sucrose was used for *sacB* gene-based counter selection in allelic exchange experiments. Primers clbP1/clbP2 and clbP3/clbP4 (Supplementary Table S3) were used for amplifying the flanking regions of the *clbP* gene. The fused flanking region (Δ*clbP*) was ligated into the *Kpn*I and *Xma*I sites of a suicide plasmid pRE112 to form the plasmid pKp-clbP1. All constructed plasmids were verified through sequencing. The suicide plasmid pKp-clbP1 (*Kp*Δ*clbP*) was conjugationally transferred from *E*. *coli* χ7213^56^ to the infant isolate *K*. *pneumoniae*. Single-crossover insertion strains were isolated on LB agar plates containing Cm. Removal of the linear suicide vector from chromosome after the second recombination between homologous regions was selected by using the *sacB*-based sucrose sensitivity counter-selection system^57^. The Cm sensitive colonies were screened and verified by PCR using a primer set clbP1/clbP4. The mutant strain was confirmed by DNA sequence analysis.

### *In vitro* infection studies

Rat small intestinal epithelial cells (IEC-6), murine duodenal epithelial (MODE-K), and mouse macrophage (RAW) cell lines were used in this study. For infections, *K*. *pneumoniae* was grown overnight in LB broth, and subcultured the following day for 2 hours. Infections were performed as previously described^14^. Cultured cells were infected (MOI: 100) for 3 hours, in triplicate, and harvested for qPCR analysis. Primary splenocytes were isolated from the spleens of specific pathogen free (SPF) WT C57Bl/6 mice as described previously^58^. Briefly, harvested spleens were freshly ground, collected with sterile D-PBS, and centrifuged (400 × g) to collect cells. To deplete red blood cells, the pellet was treated with RBC lysing buffer (0.15 M NH_4_Cl), filtered and washed with 10 mL of complete RPMI media. Splenocytes (2 × 10^6^) were seeded in 6 well plates for infection. After addition of bacteria, the plate was centrifuged (200 × g) to facilitate adhesion and incubated for three hours. RNA was isolated using Trizol and reverse-transcribed using iScript cDNA synthesis kit (BioRad, #170-8891) to generate cDNA.

### Immunostaining and cell cycle analysis

Following bacterial infection, cells were washed several times, followed by overnight gentamicin treatment. For immunostaining, cells were formalin-fixed, and permeabilized with methanol. After a one-hour block in 5% NGS, cells were incubated overnight in Phospho-γH2AX antibody (Cell signaling # 9718S). Flow cytometric analysis of Phospho-H2AX and cell cyle was performed as previously described^14,44^. Cells were stained overnight with conjugated with H2AX antibody (Alex Fluor 647 anti-H2A.X-Phosphorylated (Ser 139), Biolegend), and stained with propidium iodide. Data was collected using BD LSR Fortessa cytometer. Data analysis was performed using FCS-6 express software (De Novo Software).

### *In vivo* infection studies in mice

All mouse experiments were performed under the guidelines and approval of the Institutional Animal Care and Use Committee of the University of Florida (Protocol numbers: 20169606 and 201608025). For monoassociation studies, Germ-free (GF) *Il10*^−/−^ mice (129SvEv; n = 8/group) were transferred to gnotobiotic isolators and one week later orally gavaged with the clinical isolate *K*. *pneumoniae* (10^9^ CFU) and euthanized at 8 and 12 weeks following colonization. Stool samples from each mouse were taken every 2 weeks post-gavage and upon end point prior to euthanesia to measure bacterial colonization. For all other studies, GF *Il10*^−/−^ *or Apc*^*Min*/+^; *Il10*^−/−^ mice were transferred to SPF housing and orally gavaged with *K*. *pneumoniae* and/or SPF microbes from reconstituted cecal contents as indicated. All mice were monitored throughout the experiment for signs of distress. Stools were collected at regular intervals in liquid nitrogen and stored at −80 °C until further processing. Colonization was confirmed 1–2 weeks post-gavage via serial plating of reconstituted stool on MacConkey agar and reported as the number of colony forming units per gram (CFU/g) or via qPCR. Progression of inflammation was monitored using the Duoset Murine Lipocalin (Lcn-2) ELISA kit (R&D Systems Cat# DY1857) to measure fecal lipocalin. Upon termination of the experiment, sections of proximal and distal colon tissue were snap frozen for RNA analysis. The remaining tissue was swiss-rolled, formalin fixed and submitted for further processing. Fixed samples were paraffin-embedded and H&E stained by the UF Molecular Pathology Core. Inflammation score (0–4) was evaluated by at least one blinded independent investigator based upon histological examination of the mucosa, immune cell infiltration of the lamina propria, epithelial hyperplasia and crypt architecture as described previously^59^.

Immunostaining of formalin fixed samples for CD3 (goat anti-CD3-ε, Santa Cruz #sc-1127) was performed using Vectastain ABC (goat IgG) and M.O.M peroxidase kits (Vector labs, #NC9685284). Immune cell infiltration was assessed by quantification of CD3-positive (CD3^+^) cells per high power field (hpf, 400x). A total of 10 hpfs for each mouse, n = 4, were quantified. Results are represented as average of the mean for each mouse per indicated group.

### RNA isolation and qPCR

RNA was harvested as previously described^30^. Briefly, tissues were homogenized in Trizol reagent followed by phenol-chloroform separation. Following DNAse treatment (Turbo DNA-free kit, Ambion), cDNA was generated using iScript cDNA synthesis kit (Bio-Rad). Real-time PCR was performed on BioRad CFX-384 Real-Time PCR Detection System using SsoAdvanced Universal SYBR Green (BioRad) and primers specific for indicated genes^30^.

### qPCR array

Transcripts from the monoassociation and 20wk infection samples were analyzed using the Qiagen RT2 Profiler PCR array Mouse Inflammatory Response & Autoimmunity PAMM077ZE. 400 ng of RNA was reverse-transcribed using the RT^2^ First Strand Kit according to manufacturer protocol. Real-time PCR was performed using RT^2^ SYBR Green qPCR Mastermix. Data was analyzed using the RT2 Profiler PCR Array DATA Analysis version 3.5 (Qiagen) normalized to 3 housekeeping genes. Data was presented as fold regulation using the 2^(-Delta Delta C_T_) method and p values were calculated using Student’s t-test of replicate 2^(-Delta C_T_) values for each gene in control groups and *K*. *pneumoniae*-treated groups.

### Statistical analysis

All data were analyzed using GraphPad Prism 6, version 6.0 h (GraphPad Software, Inc., La Jolla, CA). Cell culture data was evaluated using student t-test. Mouse experiments were analyzed using the Mann-Whitney nonparametric test, one-way ANOVA, and two-way ANOVA where appropriate and as indicated.

## Supplementary information

Supplementary Material

## Data Availability

All data generated or analysed during this study are included in this published article (and its Supplementary Information files). The sequences generated are available on Pubmed via the following accession numbers: 16S determination (MK133801, MK133802, MK133803, MK133804, MK133805, MK133806) and MLST analysis (MK133349, MK133350, MK133351, MK133352, MK133353, MK133354, MK133355).
