# Supplementary material for: Microbial Colonization Coordinates the Pathogenesis of a Klebsiella pneumoniae Infant Isolate
Source: Sci Rep. 2019 Mar 4;9:3380. doi: 10.1038/s41598-019-39887-8 (PMC6399262; doi:10.1038/s41598-019-39887-8)
Supplement: Supplementary file 1 — Supplementary Material [file 41598_2019_39887_MOESM1_ESM.pdf]

Microbial Colonization Coordinates the Pathogenesis of a *Klebsiella pneumoniae* Infant Isolate

Jillian L. Pope [a], Ye Yang[a], Rachel C. Newsome [a], Wei Sun [c], Xiaolun Sun[a] , Maria Ukhanova[b], Josef Neu [a], Jean-Pierre Issa, Volker Mai [b], and Christian Jobin [a*]

Supplementary Table S1: MLST Sequence Identity

Locus	%Identity
gapA	99%
infB	100%
mdh	99%
pgi	100%
phoE	99%
rpoB	99%
tonB	99%

Supplementary Table S2. Bacterial strains and plasmids used in this study

Strains	Relevant genotype or annotation	Source or derivation
E. coli TOP10	F^- *mcrA* Δ(*mrr-hsdRMS-mcrBC*) ϕ80*lacZ*Δ*M15* Δ*lacX74* *recA1* *araD139* Δ(*ara-leu*)*7697* *galU galK rpsL endA1 nupG*	Invitrogen
E. coli χ7213	*thi-1 thr-1 leuB6 fhuA21 lacY1 glnV44* Δ*asdA4 recA1* RP4 2-Tc::Mu [λ*pir*]; Km^r	51
K. pneumoniae	Wild type strain;	This study
Δ*clbP K.pneumoniae*	*clbP* deletion	This study
**Plasmids**	**Description**	**Source**
pRE112	Suicide vector, Cm^r, mob^- (RP4)R6K *ori*, *sacB*	52
pBBR1MCS2	pSC101 *ori*, Kan^R	Provided by Dr. David Pascual
pKp-clbP1	The flanking regions of the Δ*clbP* gene were cloned into XmaI and KpnII sites of pRE112	This study
pKp-clbP2	The ORF of *clbP* gene fragment was amplified from *K. pneumoniae* and cloned into *Xma*I and *Hind*III site of pBBR1MCS2 under P_{trc} promoter	This study

Supplementary Table S3. Primers used in this study

Name	Sequence
clbPseq1-F	agccacgagttgctgcttat
clbP seq1-R	agccacgagttgctgcttat
clbP1	cgg<u>ggtacc</u>cttggcctgaaatacatactgctcataga (*Kpn*I)
clbP2	ggaacacgttagcttacacaacaaggagtgggacgatgagtaa
clbP3	cttgttgtgtaagctaacgtgttccattattgtcatcctgtga
clbP4	cgg<u>cccggg</u>gcagacgctggccggcaacagtgaactga (*Xma*I)
clbP-C1	Ataa**atg**gaacacgttagcattaaaaca *
clbP-C2	cgg<u>aagctt</u>gatattactcatcgtcccactccttgttgtgta (*Hin*dIII)

The restriction endonuclease sites are underlined. *: the bold letters show start codon of the *clbP* gene

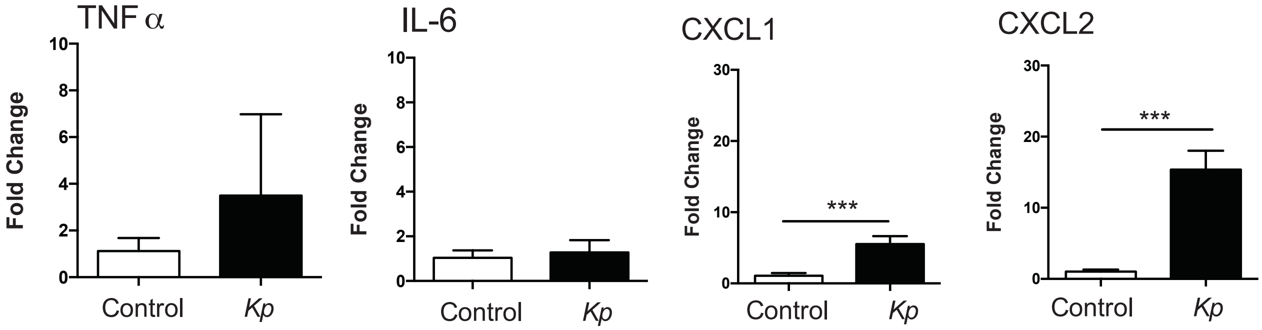

Supplementary Figure S1. ***K. pneumoniae*** **infant isolate induces inflammation in ModeK cells.** qPCR analysis of inflammatory cytokines and chemokines in ModeK epithelial cell line. Graphs represent means ± SD. ***, $P < 0.001$ (Unpaired student t-test, Welch's correction).

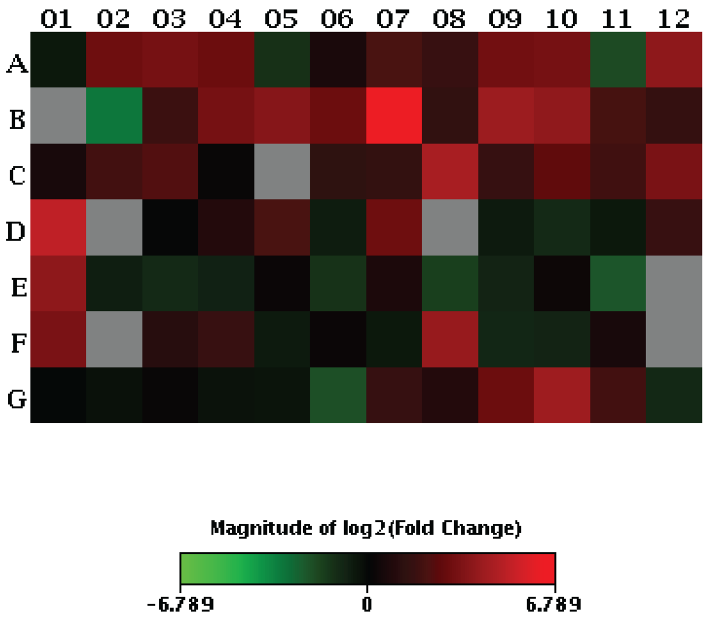

Map for Inflammation Array

	01	02	03	04	05	06	07	08	09	10	11	12
A	Bcl6	C3	C3ar1	C4b	Ccl1	Ccl11	Ccl12	Ccl17	Ccl19	Ccl2	Ccl20	Ccl22
B	Ccl24	Ccl25	Ccl3	Ccl4	Ccl5	Ccl7	Ccl8	Ccr1	Ccr2	Ccr3	Ccr4	Ccr7
C	Cd14	Cd40	Cd40lg	Cebpb	Crp	Csf1	Cxcl1	Cxcl10	Cxcl11	Cxcl2	Cxcl3	Cxcl5
D	Cxcl9	Cxcr1	Cxcr2	Cxcr4	Fasl	Fos	Ifng	Il10	Il10rb	Il17a	Il18	Il1a
E	Il1b	Il1r1	Il1rap	Il1rn	Il22	Il23a	Il23r	Il5	Il6	Il6ra	Il7	Il9
F	Itgb2	Kng1	Lta	Ltb	Ly96	Myd88	Nfkb1	Nos2	Nr3c1	Ptgs2	Ripk2	Sele
G	Tirap	Tlr1	Tlr2	Tlr3	Tlr4	Tlr5	Tlr6	Tlr7	Tlr9	Tnf	Tnfsf14	Tollip

Supplementary Figure S2. Inflammatory gene expression of 12-week *K. pneumoniae* monoassociation mice vs GF controls.

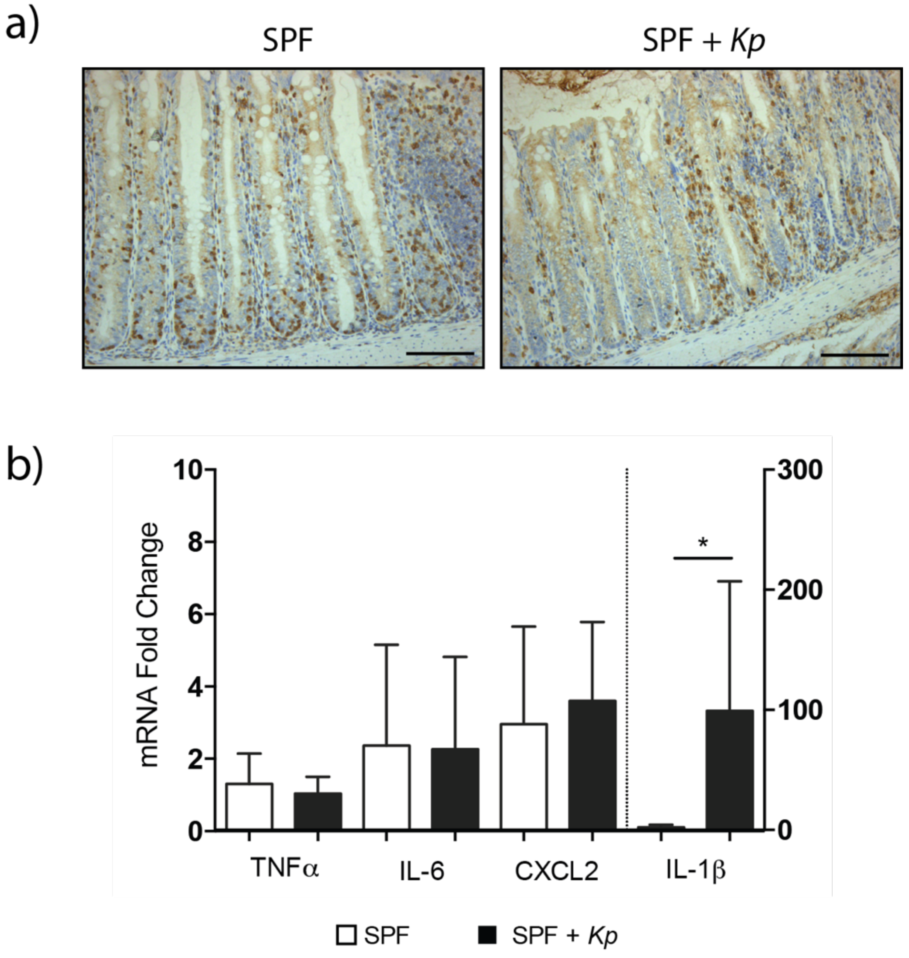

Supplementary Figure S3. Colonic inflammation in simultaneous colonization model. (a) CD3 immunostaining of representative colon tissue from SPF control and SPF *Kp* gavaged mice and (b) qPCR analysis for proinflammatory cytokines; n=3-4 per group. Graph represents mean ± SD. (Mann-Whitney t-test) *, P= 0.0317. Scale bar in bottom right corner of image is 100μm.

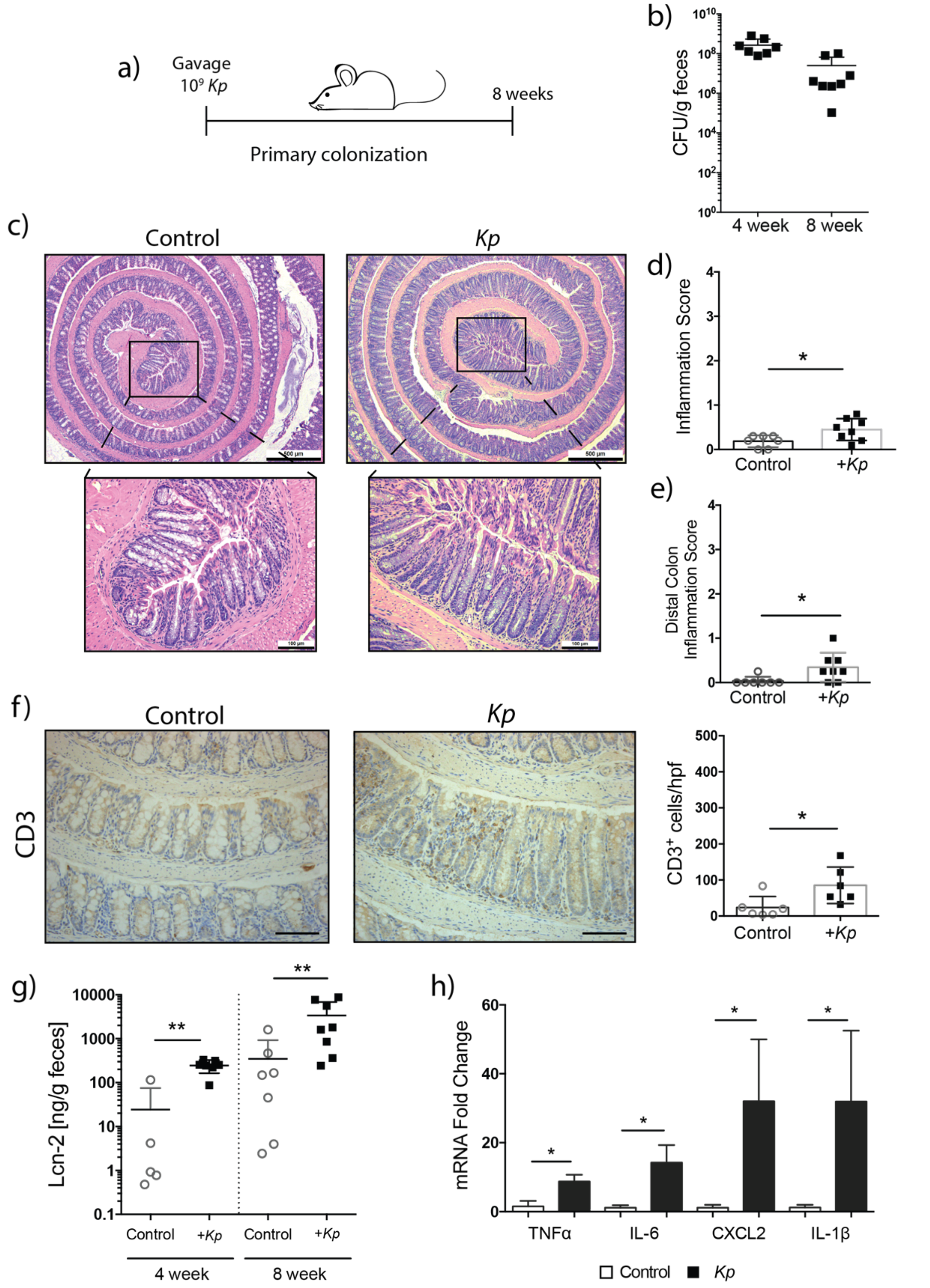

Supplementary Figure S4. *Klebsiella 51-5* induces mild colitis in presence of immature microbiota.

(a) Experiment setup. (b) *Klebsiella 51-5* fecal colonization. (c) Representative H&E stained colon tissue from control and *Kp* infected mice after 8 weeks. (d) Inflammation score of entire colon and (e) distal colon. (f) CD3 immunostaining and quantification of colon from control (n=7) and infected (n=8) mice. (f) Fecal Lcn-2 ELISA at 4 and 8 weeks post colonization of control and infected mice. (g) qPCR analysis of inflammatory cytokines of control and *Klebsiella 51*-5 infected colon tissue; n=5-6 per group. Graphs represent means ± SD. Scale bars in bottom right corner of images are 500 μm (c,f); and insets are 100 μm. *, P <0.05, **, P <0.01 (Mann-Whitney t-test)

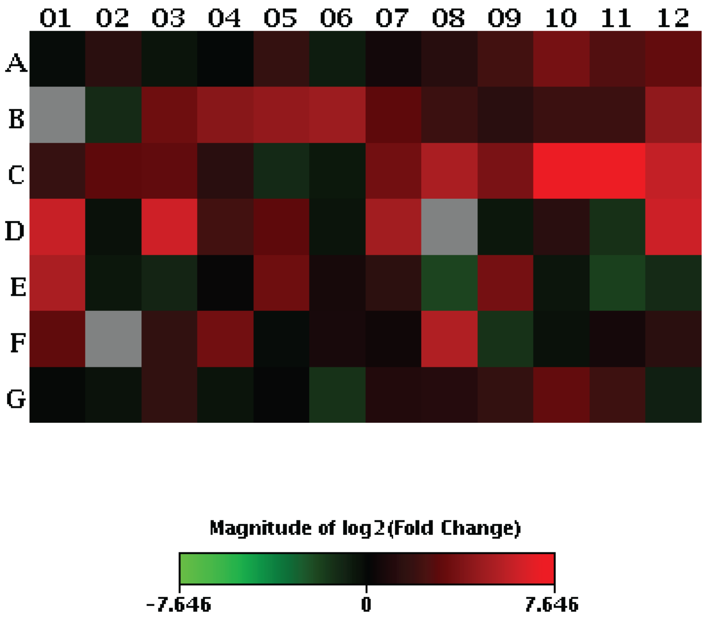

Map for Inflammation Array

	01	02	03	04	05	06	07	08	09	10	11	12
A	Bcl6	C3	C3ar1	C4b	Ccl1	Ccl11	Ccl12	Ccl17	Ccl19	Ccl2	Ccl20	Ccl22
B	Ccl24	Ccl25	Ccl3	Ccl4	Ccl5	Ccl7	Ccl8	Ccr1	Ccr2	Ccr3	Ccr4	Ccr7
C	Cd14	Cd40	Cd40lg	Cebpb	Crp	Csf1	Cxcl1	Cxcl10	Cxcl11	Cxcl2	Cxcl3	Cxcl5
D	Cxcl9	Cxcr1	Cxcr2	Cxcr4	Fasl	Fos	Ifng	Il10	Il10rb	Il17a	Il18	Il1a
E	Il1b	Il1r1	Il1rap	Il1rn	Il22	Il23a	Il23r	Il5	Il6	Il6ra	Il7	Il9
F	Itgb2	Kng1	Lta	Ltb	Ly96	Myd88	Nfkb1	Nos2	Nr3c1	Ptgs2	Ripk2	Sele
G	Tirap	Tlr1	Tlr2	Tlr3	Tlr4	Tlr5	Tlr6	Tlr7	Tlr9	Tnf	Tnfsf14	Tollip

Supplementary Figure S5. Inflammatory gene expression of *Klebsiella 51-5* infected mice vs uninfected controls for 20-week primary colonization.

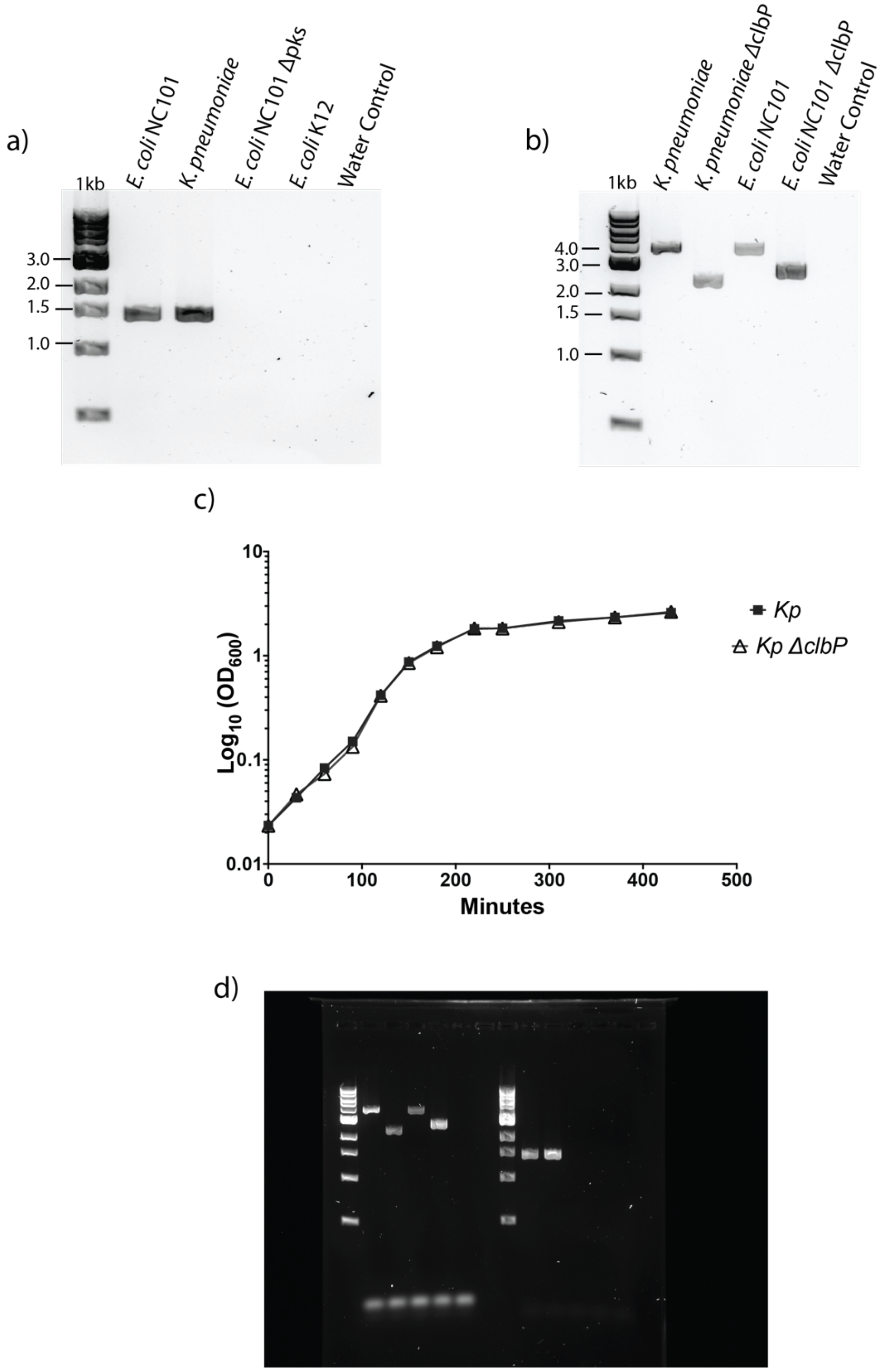

Supplementary Figure S6. Identification of the *pks* island in *Klebsiella 51-5* and deletion of clbP gene. The *pks* island was detected in *Klebsiella 51-5* (a) using R1, R2 primers (1413 bp). *E coli* NC101 was used as a positive control. NC101 Δpks and *E coli* K12 were used as negative controls, as they do not contain the *pks* island. (b) PCR anlaysis of clbP deletion using clbPseq1 primers. *Klebsiella 51-5* isolate contains full clbP sequence, including the flanking regions (3,712 bp). Deletion of clbP resulted in a 1515 bp deletion. *E.coli* NC101 and *E.coli* NC101 ΔclbP were used as controls. Both images were cropped and inverted for presentation purposes. (c) Growth curve of ΔclbP mutant vs WT *K. pneumoniae*. (d) Original gel image for panel a) and b).

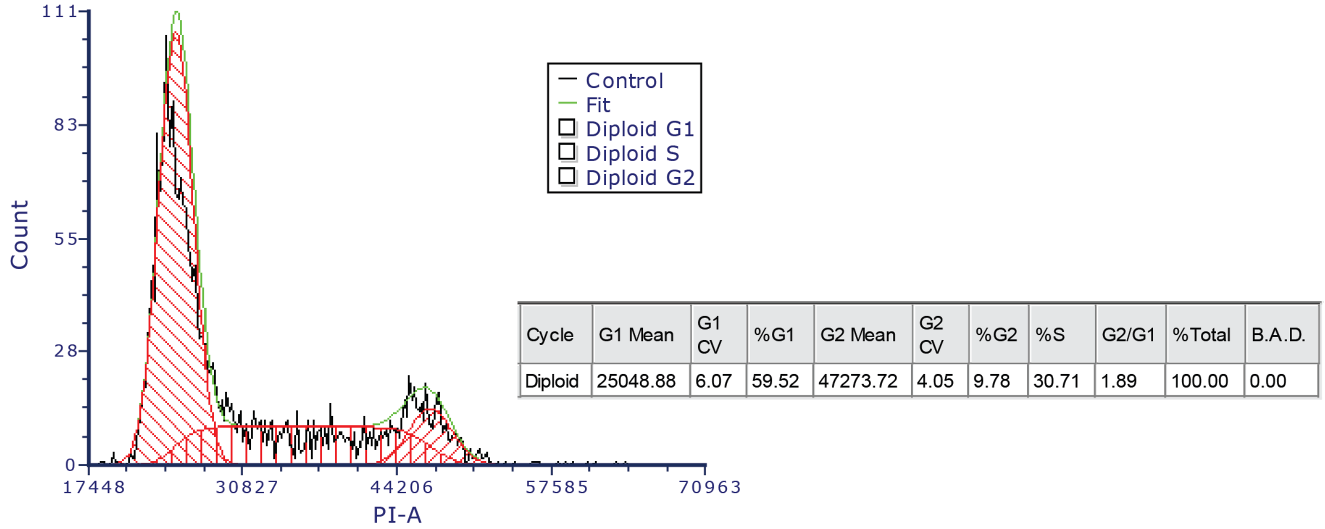

Cycle	G1 Mean	G1 CV	%G1	G2 Mean	G2 CV	%G2	%S	G2/G1	%Total	B.A.D.
Diploid	25048.88	6.07	59.52	47273.72	4.05	9.78	30.71	1.89	100.00	0.00

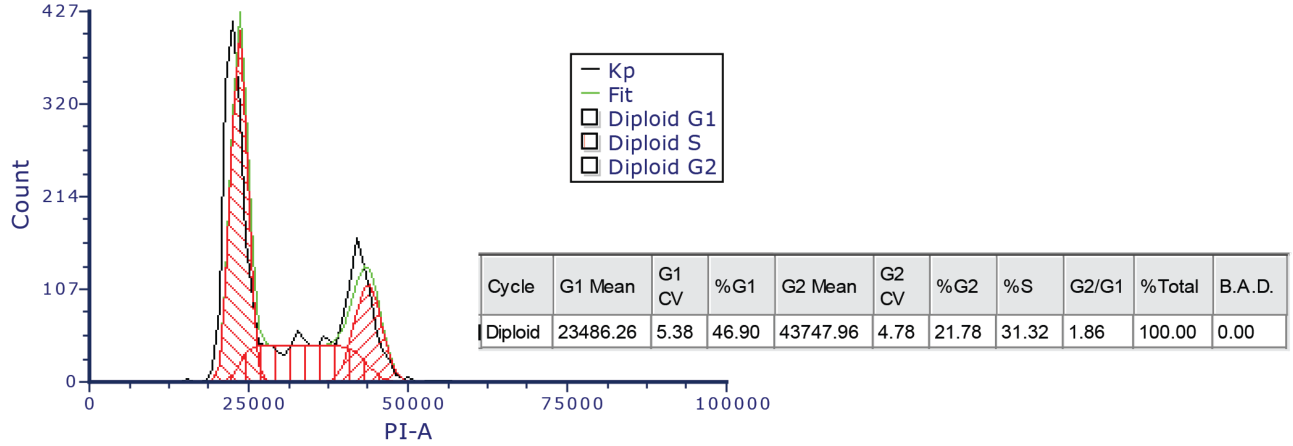

Cycle	G1 Mean	G1 CV	%G1	G2 Mean	G2 CV	%G2	%S	G2/G1	%Total	B.A.D.
Diploid	23486.26	5.38	46.90	43747.96	4.78	21.78	31.32	1.86	100.00	0.00

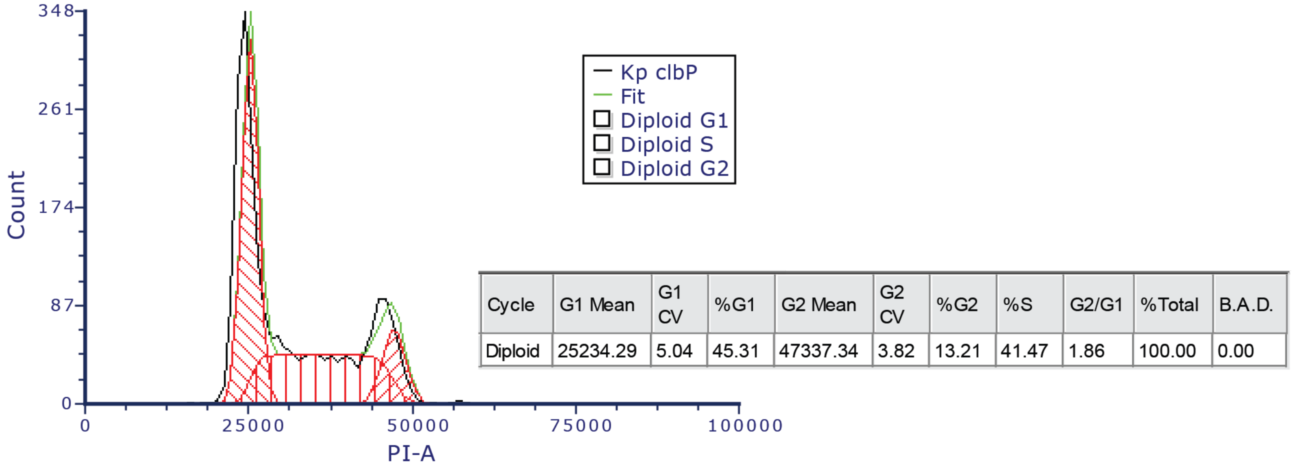

Cycle	G1 Mean	G1 CV	%G1	G2 Mean	G2 CV	%G2	%S	G2/G1	%Total	B.A.D.
Diploid	25234.29	5.04	45.31	47337.34	3.82	13.21	41.47	1.86	100.00	0.00

Supplementary Figure S7. Cell cycle analysis of IECs infected with *Klebsiella 51-5*
Cell cycle analysis was performed on singlet cells using multicycle analysis. Percentage of cells in G1 and G2 were identified and used for statistical analysis.
